# Flexible control of movement in plants

**DOI:** 10.1038/s41598-019-53118-0

**Published:** 2019-11-12

**Authors:** Silvia Guerra, Alessandro Peressotti, Francesca Peressotti, Maria Bulgheroni, Walter Baccinelli, Enrico D’Amico, Alejandra Gómez, Stefano Massaccesi, Francesco Ceccarini, Umberto Castiello

**Affiliations:** 1 Dipartimento di Psicologia Generale, Università degli studi di Padova, Padova, Italy; 2 Dipartimento di Scienze Agroalimentari, Ambientali e Animali, Università degli studi di Udine, Udine, Italy; 3 Dipartimento di Psicologia dello Sviluppo e della Socializzazione, Università degli studi di Padova, Padova, Italy; 4 Ab.Acus S.r.l, Milano, Italy

**Keywords:** Plant signalling, Behavioural ecology

## Abstract

Although plants are essentially sessile in nature, these organisms are very much in tune with their environment and are capable of a variety of movements. This may come as a surprise to many non-botanists, but not to Charles Darwin, who reported that plants do produce movements. Following Darwin’s specific interest on climbing plants, this paper will focus on the attachment mechanisms by the tendrils. We draw attention to an unsolved problem in available literature: whether during the approach phase the tendrils of climbing plants consider the structure of the support they intend to grasp and plan the movement accordingly ahead of time. Here we report the first empirical evidence that this might be the case. The three-dimensional (3D) kinematic analysis of a climbing plant (*Pisum sativum* L.) demonstrates that the plant not only perceives the support, but it scales the kinematics of tendrils’ aperture according to its thickness. When the same support is represented in two-dimensions (2D), and thus unclimbable, there is no evidence for such scaling. In these circumstances the tendrils’ kinematics resemble those observed for the condition in which no support was offered. We discuss these data in light of the evidence suggesting that plants are equipped with sensory mechanisms able to provide the necessary information to plan and control a movement.

## Introduction

Although plants are unable to move from one place to another, or perform some acts like shaking of hands as humans do, they are as much in the move as any other living organism^1,2^.

Amongst the variety of movements exhibited by plants, the oscillatory movements (also termed circumnutation), in which plants rotate around a central axis during their growth, have been widely investigated since the time of Charles Darwin^1,3–5^. In particular, Darwin’s^3^ close examination of the behaviour exhibited by climbing plants led him to widen his inquiry to other species in which he found no exception to his generalisation that circumnutation must be a universal class of plant movement^1,3–7^.

Following these pioneering observations, a number of studies on the behaviour of climbing plants, as far as support searching and attachment mechanisms are concerned, have provided insights at biomechanical, cellular and physiological level^8–23^. Less attention, instead, has been given to the form of perception at the basis of support-finding and attachment motor behaviour exhibited by climbing plants.

Our research was driven by two main questions: are climbing plants equipped with a form of perception that subtend purposeful, anticipatory behaviour as observed in a variety of animal species^24–27^? Are they able to generate a motor plan that guides an effector to a goal on the basis of the stimulus perceptual features?

To test this idea, we used kinematics to characterise the movement of circumnutation performed by the tendrils of a climbing plant (*Pisum sativum* L.) as they approach a support. Specifically, we have focused our attention on those tendrils developing in a form resembling a two-digit appendage whose movement is characterised by changes in aperture as the approach towards a support progresses. A kind of approach-to-grasp behaviour approximating that exhibited by human and non-human primates^24^ as well as by other animal species such as tetrapods^25^, birds^26^ and rodents^27^ that have evolved significant prehensile capabilities. At all cases during the course of the movement there is first a progressive opening of the appendage, followed by a gradual closure until it matches the to-be-grasped object^28^. In this respect, kinematical research has proven insightful in revealing how specific movement parameters modulate with respect to object properties, including object structure^28^. As an example from human and nonhuman primates, the velocity of hand opening during reaching is lower and the maximum aperture of the hand is smaller for thinner than for thicker stimuli^24^. In this respect, ‘thickness’ offers an ideal opportunity to parallel the ways of grasping in animal and climbing plants given that the success of grasping a support by a climber heavily depends on support diameter^7,12,15,29–31^. Differences in support thickness can determine changes in energy expenditure, which are visible on parameters characterizing the support-finding process^32^.

We reasoned that if the principles of motor planning at the basis of animal and plants approach-to-grasp behaviour are based on similar basic mechanisms, then the intrinsic properties of a support such as its thickness might have considerable effects on the kinematics of tendrils’ aperture during the approach-to-grasp behaviour.

**Figure 1:**
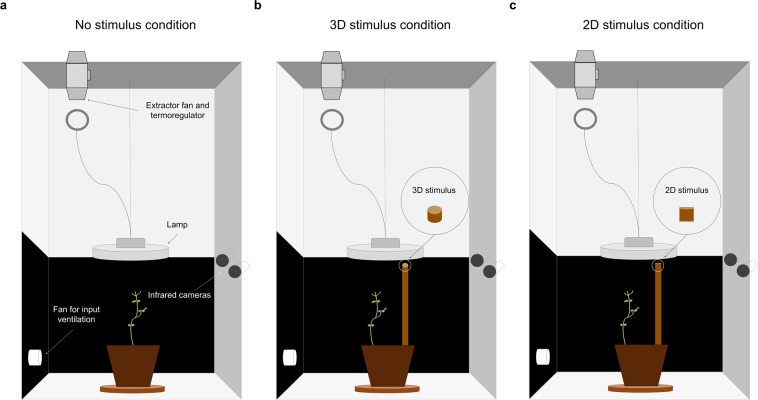
Graphical depiction of the experimental set up and the experimental conditions considered in the three experiments. (**a**) ‘No stimulus’ condition; (**b**) ‘3D Stimulus’ condition (Experiment 1) in which a wooden pole (i.e., the 3D stimulus) of 60 cm height of 1.2 cm in diameter (Experiment 1) or either 1.2. or 3 cm in diameter for Experiment 2 was positioned at a distance of 12 cm in front of the first unifoliate leaf for each plant; (**c**) ‘2D Stimulus’ condition in which the 2D representation (picture) of the 3D stimuli was attached to one of the walls of the growth chamber.

The experimental paradigm at the basis of our investigation consisted in the analysis of plant circumnutation behaviour by means of controlled time-lapse observations. In the first experiment, we video-recorded the growth of the plants in an environment lacking of any support (Fig. 1a) and in an environment in which a support, a wooden pole (i.e. the stimulus), was present (Fig. 1b).

**Figure 2:**
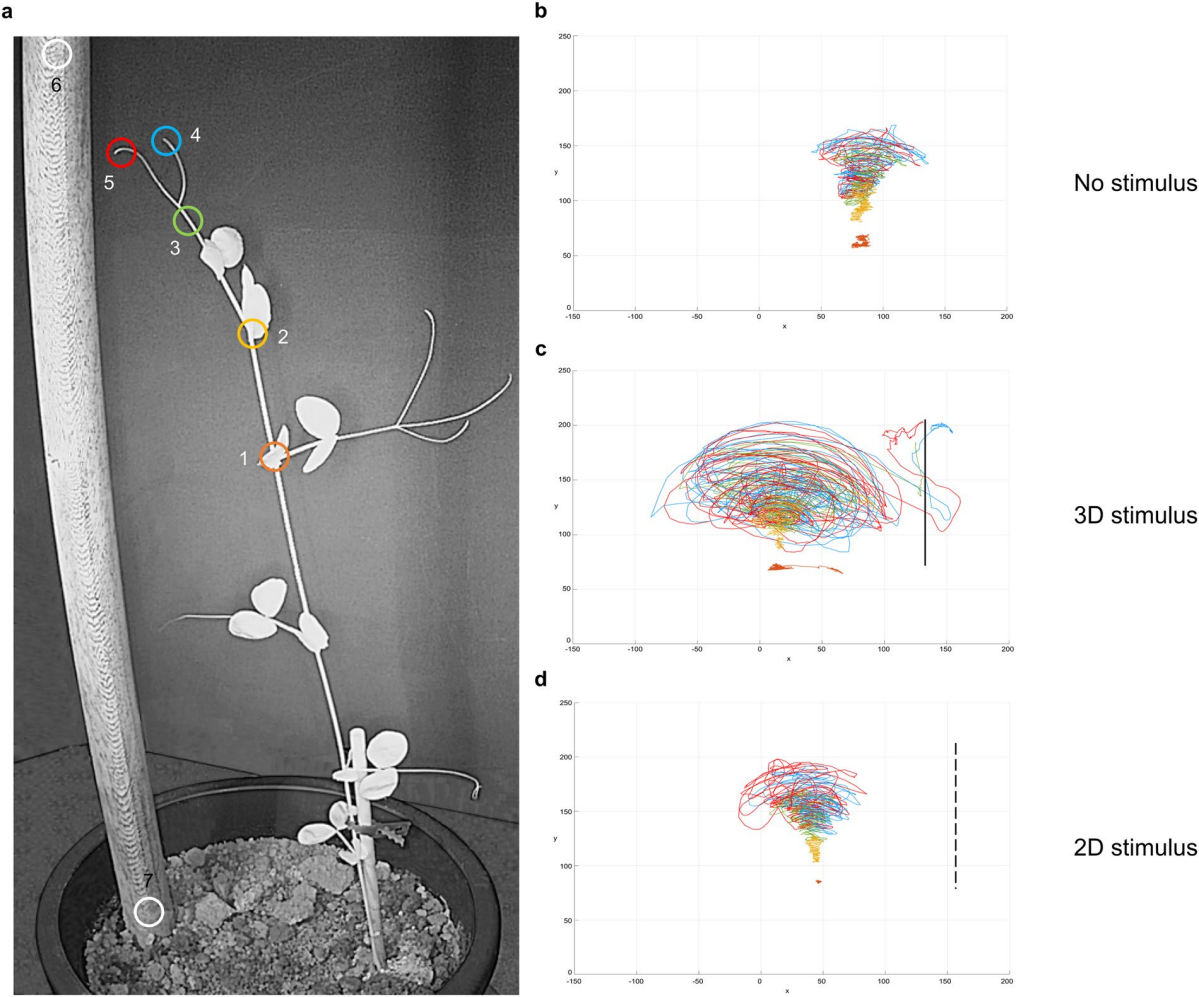
The considered landmarks of the plants and representative examples of their spatial trajectories. (**a**) Landmarks that were tracked in time through video digitalization procedures: the internode (1), the apex (2), the node below the tendrils (3), and the tips of the tendrils (4 and 5). Markers 6 and 7 were positioned upon the stimulus and served as reference points. The colours of the circles correspond to the colour of the trajectories represented in the other panels for the corresponding landmark. The projected trajectories on the vertical plane of the considered landmarks for the no stimulus (**b**), the 3D stimulus (**c**) and the 2D stimulus (**d**) conditions. The vertical line represents the 3D (solid line) and the 2D (dashed line) stimulus. Circumnutation is particularly evident for the landmark corresponding to the node below the tendrils (green line) and for the tendrils (light blue and red lines). For the apex (yellow line) circumnutation is less pronounced and directed towards the light source. When the stimulus is 3D the tendrils veered towards the stimulus and stopped at the time grasping occurred (**c**). When there is no stimulus (**b**) or the stimulus is 2D (**d**) the tendrils stop to circumnutate and remain far apart. Axis *x* = sagittal axis in mm; axis *y* = vertical axis in mm.

Video-recording was performed by means of two RGB-infrared cameras (Fig. 1) that allowed for the 3D digital reconstruction of the landmarks of interest for plant approach-to-grasp kinematics (Fig. 2; details in Methods section). We decided to consider for digitalization (i) the internode, as a reference point below the tracked leaf (Fig. 2a; marker 1/orange circle), (ii) the apex, as the index for plant growth (Fig. 2a; marker 2/yellow circle), (iii) the node below the tendrils (Fig. 2a; marker 3/green circle) and (iv) the tips of the tendrils (Fig. 2a; markers 4 and 5, blue and red circles). Because experimental models applied to animals, have already been successfully used to study plant behaviour^34–39^, we used the two-digits hand model^33^ for characterizing grasping. We equate the node below the tendrils to an hypothetical wrist that accompanies the tendrils towards the support and the tips of the tendrils to an hypothetical thumb/index finger ensemble (Fig. 2a; markers 4 and 5, blue and red circles) that during the approach phase assumes the coreography for grasping the support.

**Figure 3:**
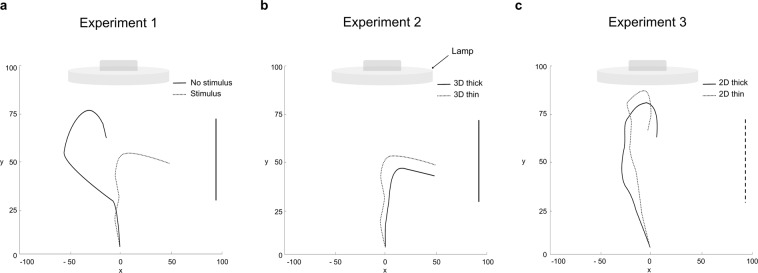
Representative examples of spatial trajectories exhibited by the apex for the different experiments. Panel (a) depicts the comparison between the 3D stimulus versus the no stimulus condition. Note that for the 3D stimulus condition the apex veered towards the support whereas for the no stimulus condition the apex grew up to a certain stage and then fell down. Panel (b) depicts the comparison between the thin and the thick 3D stimulus. For both conditions the apex veered towards the stimulus. The vertical solid line represents the 3D stimulus. Panel (c) depicts the comparison between the thin and the thick stimulus presented in 2D. For both conditions the apex grew up to a certain point and then fell down. The vertical dashed line represents the 2D stimulus. Axis *x* = sagittal axis in mm; axis *y* = vertical axis in mm.

The analysis of the spatial trajectories revealed that all the considered landmarks, except for the internode, showed a growing pattern characterised by circumnutation^1,3–6^ (Fig. 2). In particular, for the no stimulus condition, the plants circumnutate towards the light source (Figs 2b and 3a) and after various attempts to find a support in the environment, they fell to the ground (Fig. 3a). For the stimulus condition, instead, the plants moved towards the support, as if they were perceiving it (Figs 2c and 3a).

As shown in Table 1 kinematics varied significantly depending on the presence/absence of the stimulus. The average and the maximum tendrils velocity were significantly higher for the stimulus than for the no stimulus condition. The time at which the maximum tendrils velocity was reached was earlier for the no stimulus than for the stimulus condition. The maximum tendrils aperture, corresponding to the maximum distance reached by the tip of the tendrils during the approach phase, did not differ across the conditions, but the time at which the maximum distance occurred did. It occurred earlier for the stimulus than for the no stimulus condition.

**Table 1:** Kinematical and statistical values for the three experiments.

3D Stimulus vs No Stimulus	Median		*W*	*p*	*r*
	3D Stimulus	No Stimulus			
Average tendrils velocity (mm/min)	1.90	0.88	28	*0.001*	*0.57*
Maximum tendrils velocity (mm/min)	12.13	5.24	43	*0.017*	*0.43*
Time of maximum tendrils velocity (%)	83.39	30.40	25	*0.001*	*0.58*
Maximum tendrils aperture (mm)	45.63	52.12	68	*0.268*	*0.20*
Time of maximum tendrils aperture (%)	88.50	32.76	7	*0.001*	*0.62*
**3D Thin vs 3D Thick**	**Median**		***W***	***p***	***r***
	**3D Thin**	**3D Thick**			
Average tendrils velocity (mm/min)	1.90	1.21	121	*0.029*	*0.39*
Maximum tendrils velocity (mm/min)	12.13	6.65	142	*0.001*	*0.60*
Time of maximum tendrils velocity (%)	83.39	44.60	132	*0.007*	*0.49*
Maximum tendrils aperture (mm)	45.63	37.44	76	*0.044*	*0.36*
Time of maximum tendrils aperture (%)	88.50	79.87	93	*0.011*	*0.46*
**2D Thin vs 2D Thick**	**Median**		***W***	***p***	***r***
	**2D Thin**	**2D Thick**			
Average tendrils velocity (mm/min)	1.01	1.10	113	*0.500*	*0.12*
Maximum tendrils velocity (mm/min)	4.70	4.50	107	*0.341*	*0.34*
Time of maximum tendrils velocity (%)	68.10	65.14	121	*0.371*	*0.16*
Maximum tendrils aperture (mm)	55.01	57.03	102	*0.668*	*0.07*
Time of maximum tendrils aperture (%)	83.06	82.24	76	*0.760*	*0.05*

Notes. mm = millimetres; min = minutes; 3D = three dimensional; 2D = two dimensional; % = percentage of movement duration.

These findings indicate that circumnutating tendrils are able to change direction when a support is present, which is in line with preliminary observations^1,3^. We add substantially to these reports in two ways. First by providing the first full 3D kinematical characterisation of the plants’ approach-to-grasp behaviour. Second, by demonstrating that when the plants perceive the possibility for action, such as when the stimulus is detected, their pattern of growth is modified. This indicates a great deal of sophistication of plant movements as a function of the specific goal to be attained.

**Figure 4:**
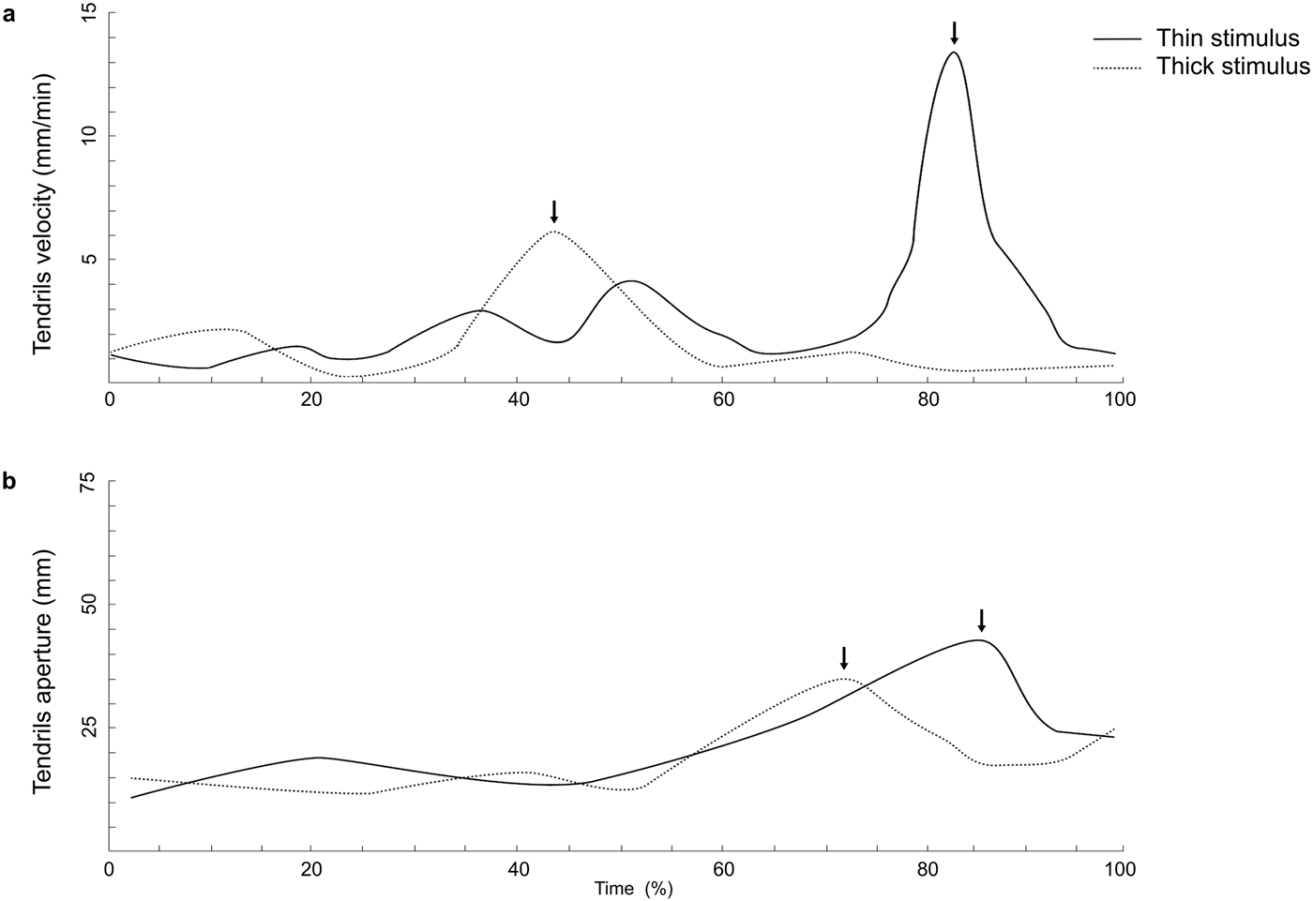
Tendrils’ kinematics is scaled with respect to the size of the stimulus. Velocity (**a**) and tendrils aperture (**b**) profiles for movements performed towards either the thick or the thin stimulus for Experiment 2. Arrows indicate the occurrence of maximum peak velocity (**a**) and maximum grip aperture (**b**) depending on stimulus thickness. Please note that when the stimulus is thicker peak velocity is anticipated and the maximum aperture of the tendrils is reached earlier for the thicker than the thinner stimulus.

Having verified that plants can program different kinematics depending on the presence of a stimulus in the environment, in Experiment 2 we characterised whether they were able to adjust kinematics depending on stimulus thickness, as happens in various animal species^24–27^. Here material, methods, data processing and analyses were exactly the same as for the first experiment (details in Methods section) except that one group of plants was tested with a ‘thick’ stimulus (3 cm diameter), whereas another group of plants was tested with a ‘thin’ stimulus (1.2 cm diameter). As witnessed by the analyses of trajectories the plants grew towards the stimulus for both stimulus conditions (Fig. 3a,b). The results not only indicate that the plants acknowledged the presence of the stimulus, but also that they scaled kinematics depending on stimulus thickness (Table 1). The peak of average and maximum tendrils velocity was higher for the thin stimulus than it was for the thicker stimulus (Fig. 4a). In temporal terms, the time at which the tendrils reached peak velocity and the time at which the tendrils reached the maximum aperture, both calculated as a percentage of movement duration were later for the thin than for the thicker stimulus (Fig. 4a,b). The maximum distance between the tendrils was significantly greater for the thin stimulus than for the thicker stimulus (Fig. 4b). This aspect is particularly important because it signifies that they extract the ‘graspable’ properties of the stimulus to determine how to engage motor modules to produce suitable behavioural outputs.

At first sight one may argue that the pattern exhibited by the plants is exactly the opposite of that showed by animals. Remember that in animal species (e.g. human and non human primates) the velocity of hand opening is lower and the maximum hand aperture is smaller for thinner than for thicker stimuli^24^. An effect ascribed to the more accurate conditions dictated by the thin stimulus. But for plants it seems to be the opposite. Previous evidence suggests that tendrils found more demanding thicker than thinner supports^32^. This is because with thicker supports the tendrils are unable to express the energy necessary to maintain tensional forces resulting in a looser attachment to the trellis^12,18,32,40,41^. In our circumstances, when the surface of the stimulus is thicker more energy might be needed for a successful attachment. Therefore the plant might apply an energy-saving strategy by slowing down circumnutation and containing the aperture of the tendrils to protect future growth conditions. For the very same reason the plants anticipate the time at which maximum tendrils’ aperture occurs so to slow down the closing phase, save energy and maximize attachment procedures. These procedures need to be done efficiently and accurately because growth-related movements are irreversible.

Before any firm conclusion can be drawn, however, it is necessary to demonstrate that are the intrinsic properties of the stimulus, such as thickness, to determine the reported effects. In Experiment 3 all was exactly the same as for the first experiment except that we used the 2D photographs of the 3D stimuli used in Experiment 2 (Fig. 1c; details in Methods section). A group of plants was exposed to the picture of the small stimulus, whereas another group was exposed to the picture of the large stimulus. From a qualitative perspective the pattern of movement mirrored exactly that observed for the condition in which no stimulus was present in the environment in Experiment 1 (Figs 2d and 3c). Most importantly, no quantitative differences in the considered dependent measures across the two stimuli were detected (Table 1).

Altogether these findings suggest that plants are equipped with a flexible control of behaviour, meaning the control of behaviour through a sensorial analysis of the environment. But, how do the plants sense the thickness of the stimulus? Although we cannot single out one particular sensory mechanism to be involved in thickness sensing during circumnutation, plants have at their disposal an array of sensory modalities^42,43^ including vision^44–46^, acoustic perception^47^, chemosensory perception^48,49^, all of which might be useful to this endeavour. Further, plant apexes are equipped with electrical, chemical, vibrational, gravitational and optical sensory transducers that permit the apex to sense the environment and provide the necessary information to plan a movement.

Therefore, in the light of this (and with a certain degree of caution) we hypothesise three possible scenarios. First, we consider a visuomotor transformation in which the visual coding of the object’s intrinsic properties (e.g., thickness) is transformed into a pattern of movement. Evidence suggests that the leaf upper and sub-epidermis comprise cells suitable to act as ocelli allowing plants to experience a sort of vision^45,46,50^. Support to this contention comes from studies on leaf mimicry in climbing plants^41^ and photoreceptor-mediated kin recognition of Arabidopsis seedlings^44^. Both of these reports seem to suggest that plants are able to gather information about their environmental setting through vision-based inputs and behave accordingly.

The second scenario implies that in order to acquire information about the surroundings plants relies on chemoreception of volatiles^43,51^. The tendrils might be able to sense the properties of the stimuli via chemical cues. In this respect it is well known that some plants use volatile blends to locate several host plants^52,53^. Furthermore, recent literature, using modern analytical techniques to visualise the chemical environment of plants, has unravelled mechanisms of plant-environment interaction that might serve to adapt to an ever-changing environment^49^.

The third scenario considers echolocation. In this perspective, a plant could emit sonic clicks and capture the returning echoes as to get information regarding the surroundings^54,55^. This could be an effective manner for climbing plants to locate suitable supports to attach to. Indeed supports of different structural properties reflect or absorb an acoustic wave in different manners, thus determining the extent and vividness of echoes rebounding and the perceived affordance a given structure delivers to the plant. This might allow the plant to put in place the kind of behaviours as those described here.

To wrap up, there might well be structures that mediate reliable detection of ethologically relevant sensory features. Recruitment of such hypothetical assemblies appears to link perception to action by furnishing the motor commands with the information necessary to release an appropriate response. Information about the properties of objects might be important for accomplishing the goal of the action. In this respect provision of an unsuitable smooth support for climbers such as a glass rod leads to some initial winding followed by unwinding and continuation of the search for a suitable support elsewhere^1,3^. We add to this observation suggesting that detection of unsuitable supports can occur well before any mechanical stimulation from impact, as our experiment with 2D photographs seem to demonstrate.

Altogether, these findings have considerable implications for our understanding of plant physiology, with particular importance for the kind of signals that drive movement in plants and how they are detected. Collectively, our observations raise questions at an evolutionary level about the ubiquity of this mechanism in other species. Despite plants and animals are two very unique evolutionary adaptations for multicellular life, each depending on unique kingdom-specific sets of cells, tissues and organs, they might have evolved signalling networks and mechanisms based on a common tool-set from our unicellular common ancestor.

## Methods

### Experiment 1: Subjects

Twenty-five snow peas (*Pisum sativum* var. saccharatum cv Carouby de Maussane) were chosen as model plants. Healthy-looking pea seeds were selected, potted and kept at the conditions outlined below. Plants were randomly assigned to each experimental condition.

### Experiment 1: Germination and growth conditions

Cylindrical pots (D 20 cm height 20 cm) were filled with agricultural soil and sowed with 1 seed per pot by placing the seed at a distance of 6 cm from pot centre and sowing depth of 2.5 cm. Individual pots were then enclosed in growth chambers (Cultibox SG combi 80 × 80 × 160 cm) for germination and growth in controlled environment conditions. Chamber air temperature was set at 26 C by an extractor fan equipped with a thermo-regulator (TT125; 125 mm-diameter; max 280 MC/H vents) and an input-ventilation fan (Blauberg Tubo 100–102m^3^/h). The combination of the two fans allowed for a steady air circulation into the growth camber with an air mean residence time of 60 seconds. The disposition of the fan was such that air circulation did not affect the natural plants’ movements. Plants were grown with a 11.25- hour photoperiod (5.45 am to 5 pm) under a cool white led lamp (V-TAC innovative LED lighting, VT-911–100W, Des Moines, IA, USA) that was exactly centred at 50 cm above each seedling. Photosynthetic Photon Flux Density at 50 cm under the lamp in correspondence of the seedling was 350 umolPh m-2 s-1 (quantum sensor LI-190R, Lincoln, Nebraska USA). Reflective Mylar® film of chamber walls allowed for better uniformity in light distribution. Pots were watered with tap water as needed three times a week. Experimental treatments were applied to single plants while individually growing in one growing chamber. Treatments were replicated five times by randomly assigning treatments to the four growing chambers.

### Experiment 1: Stimulus

The stimulus was a wooden pole of 60 cm height of either 3 cm in diameter positioned at a distance of 12 cm in front of the first unifoliate leaf for each plant.

### Experiment 1: Video recording and data analysis

For each growth chamber, a pair of cameras RGB-infrared cameras (i.e., IP 2.1 Mpx outdoor varifocal IR 1080 P) were placed 110 cm off the ground, spaced at a distance of 45 cm to record stereo images of the plant. The cameras were connected through Ethernet cables to a 10-port wireless router (i.e., D-link Dsr-250n) connected via Wi-Fi to a PC on which the frames acquisition and saving process was controlled by means of CamRecorder software (Ab.Acus s.r.l., Milan, Italy). To maximise contrast between pea anatomical landmarks (e.g., tendrils) and the background for the sake of recording, black felt velvet was fixed on some sectors of the growth boxes walls and the wooden stimuli were darkened with charcoal. The intrinsic, extrinsic and the lens distortion parameters of each camera were estimated using Matlab Camera Calibrator app. The images data-set used for the single-camera parameters extraction process was created by taking 20 pictures of a chessboard (squares’ side 18 mm, 10 columns, 7 rows) from multiple angles and distances, in natural non-direct light conditions. For stereo calibration, the same chessboard used for the single camera calibration process was placed in the middle of the growth chamber. Then, a picture was taken by the two cameras, to extract the stereo calibration parameters. In the experimental protocol, a frame was acquired every 3 minutes (frequency 0.0056 Hz) synchronously from each camera of the growth chamber. An ad hoc software (Ab.Acus s.r.l., Milan, Italy) developed in Matlab was used to position the markers, track their position frame-by-frame on the images acquired by the two cameras associated with each plant, and to reconstruct the 3D trajectory of each marker. The initial frame was defined as the frame at which the tendrils of the coiled leaf were visible from the apex. The end of the plant movement was defined as the moment in which the tendrils of the leaf started to coil the stimulus (stimulus condition) or the frame before the fall of the plant for the no stimulus condition. Markers were inserted post-hoc on the anatomical landmarks of interest, namely the internode, the apex, the node below the tendrils, and the tips of the tendrils. Markers were also positioned upon the stimulus and served as reference points. Tracking procedures were performed at first automatically throughout the time course of the movement sequence using Kanade-Lucas-Tomasi (KLT) algorithm on the frames acquired by each camera, after distortion removal. The tracking was manually verified by the experimenter, who checked the position of the markers frame-by-frame. The 3-D trajectory of each tracked marker was computed by triangulating the 2-D trajectories obtained from the two cameras. The dependent measures specifically tailored to test our experimental hypothesis were: (i) the spatial trajectories of the considered landmarks; (ii) the average and the maximum velocity of the tendrils during circumnutation; (iii) the maximum tendrils aperture, corresponding to the maximum distance reached by the tip of the tendrils during the approach phase; and (iv) the time at which the maximum aperture of the tendrils occurred. These measures were chosen because they correspond to the key kinematical landmarks used to characterise reaching to grasp movement in a variety of animal species^24^.

### Statistics

Statistical tests comparing the median value for each of the considered dependent measure across conditions (Experiment 1: stimulus vs no stimulus; Experiment 2: 3D thick vs 3D thin stimulus; Experiment 3: 2D thick vs 2D thin stimulus) were performed using the Wilcoxon rank sum test (one-tailed). In addition to W-statistic and the p-value, we report the effect’s size calculated as r = z/√N, in which z is the z-score and N is the total number of observations^56^. Statistical analyses were run using the computing environment R^57^, and the function wilcox.test.

### Experiment 2

Methods, procedures and data analysis are exactly the same adopted for Experiment 1 except that one group of plants was tested with a thick stimulus (3 cm diameter), whereas another group of plants was tested with a thin stimulus (1.2 cm diameter).

### Experiment 3

Methods, procedures and data analysis are exactly the same adopted for Experiment 1 except that we used the 2D photographs of the 3D stimuli used in the previous experiment. A group of plants was exposed to the picture of the thin stimulus, whereas another group was exposed to the picture of the thick stimulus.

## Data Availability

The datasets generated during and/or analysed during the current study are available at https://osf.io/ncj54/.
